# Biodeterioration Control of the Longhu Pagoda Stone Heritage, China: Identification of Deteriogens and Evaluation of Biocidal Efficacy

**DOI:** 10.3390/ma19163361

**Published:** 2026-08-07

**Authors:** Juanli Wang, Ye Tian, Jiayao Tian, Qingyang Xie

**Affiliations:** Key Laboratory of Archaeological Exploration and Cultural Heritage Conservation Technology, Ministry of Education, Institute of Culture and Heritage, Northwestern Polytechnical University, Xi’an 710072, China; tianye08192024@163.com (Y.T.); 18791731988@163.com (J.T.); 18735967040@163.com (Q.X.)

**Keywords:** stone cultural heritage, Longhu Pagoda, biodeterioration, bryophytes, lichens, biocides

## Abstract

Longhu Pagoda, a national key cultural heritage site located at the southern foot of Hushan Mountain in Liubao, Jinan, Shandong Province, is a masterpiece of Tang-dynasty stone pagoda architecture and a core component of the Shentong Temple heritage complex alongside the Four Gates Pagoda. The pagoda features a distinctive “Tang-dynasty body, Song-dynasty crown” structure: its stone base and main body, carved with intricate high-reliefs of dragons, tigers, heavenly kings, arhats, and apsaras, date back to the late Tang Dynasty (717–845 AD), while the brick eaves and roof were reconstructed during the Northern Song Dynasty. Severely threatened by biodeterioration induced by bryophytes, lichens, and associated microorganisms, the pagoda’s exquisitely carved stone surface has undergone irreversible aesthetic degradation and structural weakening, threatening its thousand-year-old artistic integrity. Biocide treatment is a commonly adopted strategy for stone heritage conservation. This study aims to characterize the specific biodeteriogens colonizing Longhu Pagoda and evaluate the efficacy of various biocides for targeted conservation intervention. In this study, biological samples collected from Longhu Pagoda were first identified: bryophyte samples contained chloroplasts, with some having morphological characteristics similar to Pottiaceae or Grimmiaceae; symbiotic algae isolated from lichen samples were identified as *Chlorella* sp.; four dominant fungal strains were isolated via morphological observation and molecular sequencing (ITS), namely *Aspergillus niger* (F1), *Trichoderma yunnanense* (F2), *Talaromyces ruber* (F3), and *Aspergillus aflatoxiformans* (F4). On this basis, the inhibitory effects of different biocides on dominant deteriorating organisms were systematically evaluated via chlorophyll fluorescence analysis, algal growth inhibition tests, and oxford cup assays, combined with field verification on Longhu Pagoda. The optimal biocide with stable inhibition efficacy and favorable stone compatibility was screened out. The results provide technical support for the biological conservation of Longhu Pagoda and similar stone cultural relics suffering from microbial biodeterioration.

## 1. Introduction

Stone monuments are precious cultural heritage that carry historical civilization, artistic value and technological wisdom of humanity. Open-air stone monuments in China, including ancient pagodas, grottoes and cliff sculptures, are constantly affected by natural weathering, and biodeterioration has become one of the most critical factors threatening their long-term preservation [1,2,3,4]. As the most common epilithic organisms, bryophytes and lichens easily colonize and spread on the surface of cultural relics under suitable temperature, humidity and nutrient conditions, forming continuous biological coverage. This not only destroys the aesthetic integrity and historical features of cultural relics, but also causes irreversible damage to stone materials through physical mechanical destruction and chemical corrosion [5,6].

Lichens are stable symbiotic complexes of fungi, green algae and cyanobacteria. Their mycelium can penetrate into the micro-cracks and pores of stone materials, producing physical expansion effect, enlarging cracks and reducing the structural strength of stone. Meanwhile, organic acids such as oxalic acid and citric acid metabolized by symbiotic fungi and bryophytes continuously corrode carbonate and silicate minerals, leading to mineral ion dissolution and structural disintegration [7,8]. In addition, the biological cover significantly retains surface moisture, aggravates salt migration, wet-dry cycles and freeze–thaw damage inside the stone, and further induces the colonization of secondary microorganisms such as bacteria and molds, forming a vicious cycle of “biological colonization–moisture enrichment–chemical corrosion–structural damage” [9,10,11].

At present, the mitigation of microbial contamination on stone cultural relics mainly relies on chemical, physical, and mechanical approaches, each of which possesses distinct advantages and inherent limitations [12]. Compared with physical cleaning and mechanical polishing methods, chemical biocides have become the most prevalent technique for the field treatment of moss, lichen, and fungal deterioration of stone heritage, due to their convenient operation and rapid effectiveness [13,14]. In particular, chemical biocides dominated by quaternary ammonium salts and isothiazolinones are widely applied in the prevention and control of biological deterioration of cultural relics, benefiting from their favorable economic efficiency and stable antimicrobial performance. Such treatments exhibit good adaptability to various stone substrates and are suitable for large-scale disinfection of massive stone heritage structures, including ancient pagodas and large stone carvings [15,16]. Sanmartín et al. [17] evaluated the removal efficiency of algal biofilms on granite buildings using ethanol–water mixture (1:1), benzalkonium chloride, and several commercial quaternary ammonium salt-based biocides (Biotin^®^, Biotin T, Preventol^®^ RI80, New Des 50). Their results verified significant differences in treatment efficacy and stone substrate compatibility among various commercial biocide products. Li et al. [18] systematically assessed the antifungal and antibacterial performance of four typical biocides, including benzalkonium chloride, isothiazolinone, penconazole, and thiabendazole, against dominant microorganisms on limestone relics. The study highlighted that environmental factors such as rainfall can dilute biocide concentrations and weaken treatment efficiency, leading to the rapid recolonization of microorganisms, which substantially restricts the long-term protective performance of chemical treatments. Furthermore, Zhang et al. [19], Li et al. [2], and Sierra-Fernandez et al. [4] investigated the inhibition mechanisms and application performances of isothiazolinones, triazoles, and benzimidazoles against typical microorganisms colonizing stone relics. Considerable differences in functional targets, antimicrobial mechanisms, and treatment effectiveness were confirmed among different categories of biocides. Overall, biocides differ greatly in their action mechanisms, microbial inhibition efficiency, and stone compatibility. Therefore, targeted biocide screening and performance evaluation are essential for reliable heritage conservation. To achieve safe, efficient, and low-damage biological treatment of stone relics, it is necessary to clarify the inhibitory characteristics of different biocides against dominant deteriorating microorganisms and screen the optimal effective concentration before field application. Such strategies can ensure satisfactory antimicrobial effects while minimizing the secondary damage caused by chemical agents to stone substrates [20].

Longhu Pagoda, located in Jinan, Shandong Province, China (36°36′32″ N, 117°02′41″ E), is a national key protected stone cultural heritage site and a typical representative single-layer stone pagoda with exquisite high-relief stone carvings dating back to the Tang Dynasty in northern China. Renowned for its delicate dragon and tiger reliefs, it serves as a crucial material for the research of ancient Chinese stone architecture and Buddhist art [21]. Having been exposed to the natural environment for over 1000 years and subjected to routine modern repairs and reinforcement, the pagoda retains well-preserved original stone structures and surface decorative carvings. However, the long-term influence of local humid and semi-humid climate, accompanied by seasonal rainfall, fluctuating temperatures, persistent high humidity and wind erosion, has provided favorable conditions for extensive biological colonization on the stone surface, resulting in severe biodeterioration. Typical biological alterations widely observed on the pagoda surface include dense lichen crusts, continuous moss carpets, scattered fungal mycelia and widespread biological patina. Long-term proliferation of these biodeteriogens further triggers severe stone damages such as biopitting, surface discoloration, grain detachment and micro-cracking, which progressively undermine the structural integrity and aesthetic value of this ancient stone relic. Therefore, it is urgent to carry out targeted research on the biological deterioration mechanism and scientific prevention and control technology of Longhu Pagoda to curb ongoing stone degradation and realize the long-term sustainable protection of the relic.

Accordingly, this study focuses on biological deterioration occurring on the surface of the Longhu Pagoda. Lichen-associated fungi and related microbial communities were accurately identified via morphological observation and molecular sequencing (ITS). Three common biocides used in cultural heritage conservation, namely quaternary ammonium compounds, benzalkonium chloride, and ketone-based corrosion inhibitors, were selected for laboratory simulations and field tests. Using chlorophyll fluorescence measurement, algae growth inhibition assays, and the oxford cup method, the inhibitory effects of these agents against bryophytes, green algae, cyanobacteria, and lichen-associated fungi were systematically compared. The optimal biocide was screened out, and its safe and applicable concentration was determined. This study can provide direct technical support for the scientific control of biological deterioration at the Longhu Pagoda and also offer a theoretical basis and practical reference for the control of biological decay and preventive conservation of similar open-air stone cultural relics.

## 2. Materials and Methods

### 2.1. Material

Sodium chloride (NaCl, analytical grade) was purchased from Sinopharm Group (Shanghai, China). Agar powder (microbial grade) was purchased from Aladdin Biochemical Technology Co., Ltd. (Shanghai, China). Antibiotic–antimycotic solution (100×, Cat. No. A6533) and Tween 80 (Cat. No. T8194) were both obtained from Macklin Biochemical Co., Ltd. (Shanghai, China). Potato Dextrose Agar (PDA, Cat. No. AC13825) and Potato Dextrose Broth (PDB, Cat. No. AC13840), as well as propidium iodide (PI, ≥95%, Cat. No. P72320), were supplied by Acmec Biochemical Co., Ltd. (Shanghai, China). All media were sterilized by autoclaving at 121 °C for 15 min and stored at 4 °C until use. BG11 medium (powder, for cyanobacteria culture, Cat. No. PM1220) was purchased from Coolaber Technology Co., Ltd. (Beijing, China). BBM medium (powder, for green algae culture, Cat. No. HB0387) was purchased from Hopebio Co., Ltd. (Qingdao, China). Both media were prepared according to the manufacturer’s instructions and autoclaved at 121 °C for 15 min before use. For symbiotic algae isolation, BG11 and BBM media were supplemented with 100× antibiotic cocktail (Solarbio Science & Technology Co., Ltd., Beijing, China, Cat. No. A8150) containing 10 mg/mL ampicillin, 10 mg/mL streptomycin and 10 mg/mL kanamycin at a dilution of 1:100 to prevent microbial contamination. Additional 20.0 g/L agar was added to prepare solid media. Benzalkonium chloride (purity ≥ 99%, Cat. No. BC9901) was obtained from Tianjin Kewei Jinhong Environmental Protection Technology Co., Ltd. (Tangshan, China). Total Algae Control was purchased from Brandt Consolidated, Inc. (Springfield, IL, USA). Dodecyldimethylbenzyl ammonium chloride (1227) was supplied by Changzhou Tianhe Chemical Factory Co., Ltd. (Changzhou, China). PCR Clean-up Kit (Cat. No. D6402-01) was obtained from Omega Bio-tek (Norcross, GA, USA). Sanger sequencing was performed by Sangon Biotech Co., Ltd. (Shanghai, China). The experimental stone samples were collected from the mountainous area near the Longhu Pagoda in Licheng District, Jinan, Shandong Province, China. These samples have the same origin and lithology as the original building stone of the pagoda, which is a typical high-quality bluestone from the southern mountainous region of Jinan.

### 2.2. Main Instruments

Ultra-depth three-dimensional morphological observation of microbial biofilms on stone relic surfaces was performed using a KEYENCE VHX-2000 digital microscope (KEYENCE Corporation, Osaka, Japan). All aseptic microbial operations were carried out in a laminar flow cabinet (SW-CJ-1FB, Suzhou Purification Equipment Co., Ltd., Suzhou, China). Fungal and algal strain cultivation and incubation were completed in a biochemical incubator (LRH-250, Yiheng Scientific Instrument Co., Ltd., Shanghai, China). Experimental utensils were sterilized using an autoclave (GI54T, ZEALWAY Instrument Co., Ltd., Xiamen, China). An electronic balance (PX224ZH, OHAUS Corporation, Parsippany, NJ, USA) was used for precise reagent weighing. Sample mixing and homogenization were performed with a Vortex-Genie 2 vortex mixer (Scientific Industries, Bohemia, NY, USA). A digital caliper (MNT919010, Meinart Tool Co., Ltd., Changzhou, China) was applied to accurately measure the radius of antibacterial inhibition zones. In addition, chlorophyll fluorescence parameters were tested via a Yaxin-1162 chlorophyll fluorometer (Yaxin Liyi Technology Co., Ltd., Beijing, China), and the in vivo physiological activity of algae was assessed using an AquaPen-C AP110-C handheld algae fluorometer (PSI, Drásov, Czech Republic).

### 2.3. Sampling Site and Sampling

**Figure 1:**
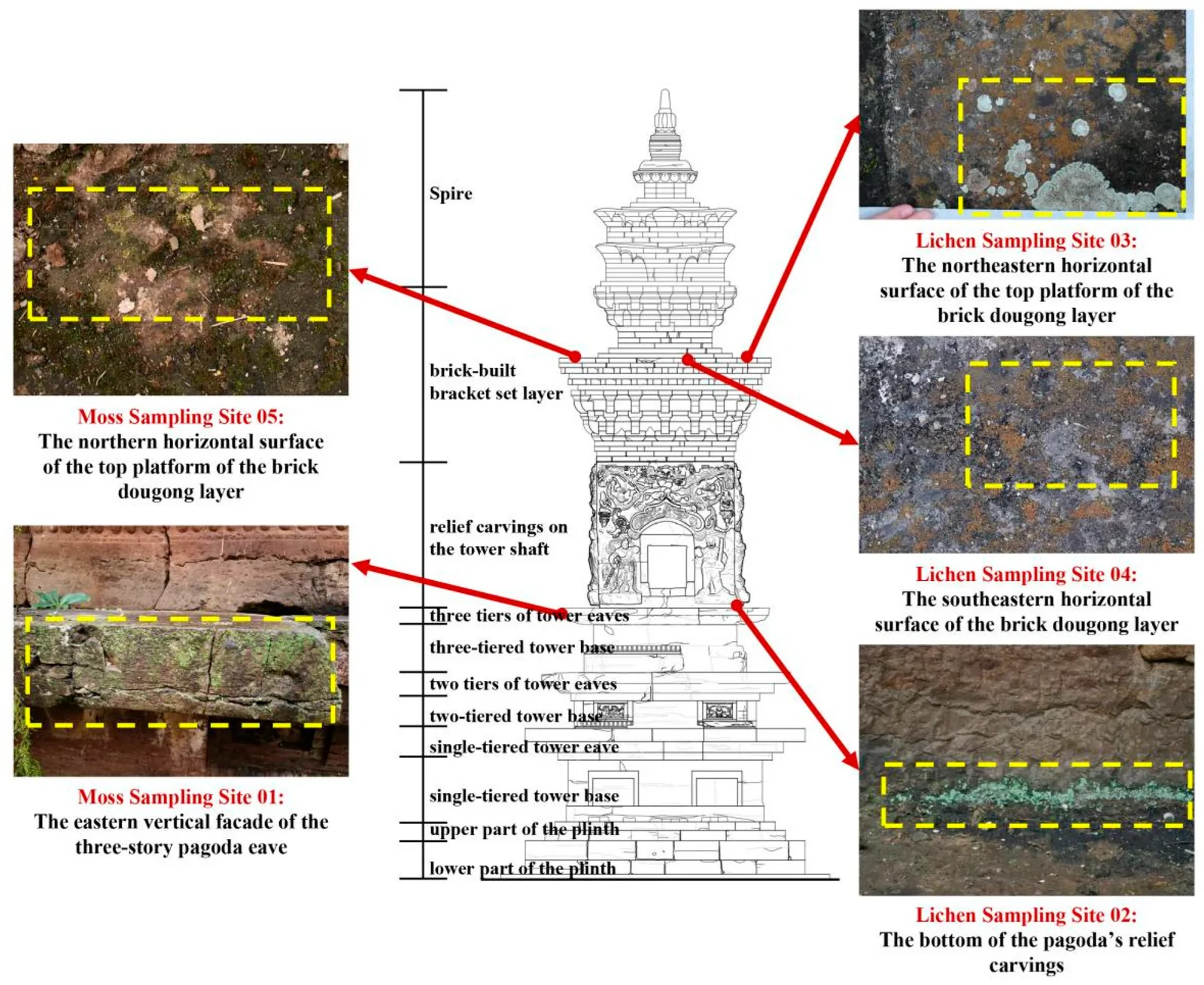
Sampling points for moss and lichen on the longhu pagoda. The yellow dashed-line boxes denote the actual sampling areas for biological material collection.

Representative sampling is the prerequisite for accurately analyzing biodeterioration characteristics of stone cultural relics. In this study, sampling sites were comprehensively selected based on the overall biological coverage, deterioration degree, and spatial distribution characteristics of Longhu Pagoda stone surfaces, covering different orientations, heights, and severely deteriorated areas to ensure maximum representativeness and universality of the collected samples. In this study, a total of five biological samples were collected from the Longhu Pagoda. Among them, samples 01 and 05 were bryophytes, and samples 02, 03, and 04 were lichens. Sample 01 was obtained from the eastern vertical facade of the three-story pagoda eave. Sample 05 was collected from the northern horizontal surface of the top platform of the brick dougong layer. Sample 02 was taken at the bottom of the pagoda’s relief carvings. Sample 03 was acquired from the northeastern horizontal surface of the top platform of the brick dougong layer, and sample 04 from the southeastern horizontal surface of the same platform. The detailed sampling locations are shown in Figure 1.

Considering the different growth forms of stone biodeteriogens, classified and targeted sampling methods were adopted to ensure sampling accuracy. Specifically, moss and lichen colonies, which form macroscopic thallus structures on stone surfaces, were collected using sterile scalpels and tweezers to obtain complete tissue samples, ensuring the integrity of fungal and algal symbionts. For thin surface biofilms and residual epilithic microorganisms that cannot be physically stripped, sterile cotton swabs were used for surface wiping sampling. All samples were taken from discolored and biologically colonized stone surfaces of Longhu Pagoda, immediately placed into sterile centrifuge tubes and sample bags; stored in a low-temperature incubator at 4 °C during transportation.

In the laboratory, intact bryophyte individuals and lichen thalli were carefully selected, cleaned, and inoculated onto PDA, BG11, and BBM media for isolation and purification. All media were supplemented with a mixed antibiotic cocktail (ampicillin, streptomycin, kanamycin; 100 μg mL^−1^ for each) to prevent bacterial contamination.

All samples were incubated at 25 °C for 7–14 days. Fungal and algal suspensions were prepared from individual colonies in spore buffer (0.9% (*m*/*v*) NaCl and 0.01% (*v*/*v*) Tween 80). Then 5 μL of the suspensions were spotted onto the center of PDA, BG11, and BBM plates and incubated at 25 °C for morphological observation and purification. All purified isolates were maintained on corresponding agar slants at 4 °C for subsequent experiments.

### 2.4. Experimental Procedures

#### 2.4.1. Isolation and Culture of Lichen Symbionts

Lichen symbionts were isolated using the tissue isolation method. Lichen thalli were cleaned to remove surface dust and impurities, then soaked in 1.5 mL tubes and washed with sterile water for 5 min with vortexing. This washing step was repeated 1–2 times. Thalli were surface-sterilized in 75% ethanol for 2 min, then rinsed twice with sterile water for 5 min each. The algal layer was separated, ground in sterile water, diluted, and spread on BG11 and BBM agar, then incubated at 25 °C under a 14 h:10 h light/dark cycle at 3000 lux. Medullary tissues were fragmented and inoculated on PDA medium at 25 °C in darkness. Visible colonies and algal clusters were picked and subcultured repeatedly on corresponding media to obtain pure isolates.

#### 2.4.2. Micromorphology Observation of Bryophytes

Intact individual plants were carefully excised from bryophyte samples using sterile forceps, rinsed gently in sterile water to remove surface impurities, and placed in a Petri dish containing deionized water. A clean glass slide was prepared with 1–2 drops of deionized water, and the cleaned bryophyte tissues were placed onto the slide. A coverslip was placed over the sample and gently pressed to flatten the tissues. The slides were immediately observed using an ultra-depth three-dimensional microscope (VHX-2000, KEYENCE, Osaka, Japan), and representative microscopic images were captured for morphological analysis.

#### 2.4.3. Bryophyte Killing Assay

In the bryophyte killing assay, healthy moss samples were placed in 9 cm Petri dishes. The three biocides were diluted with sterile deionized water to working concentrations of 1%, 3%, and 5% (*v*/*v*). A solvent blank control (CK) treated with an equal volume of sterile deionized water (the dilution solvent) was established in parallel to exclude solvent interference. Each dish was uniformly sprayed with 1–1.5 mL of the corresponding solution and incubated for 24 h. Chlorophyll fluorescence was measured at an actinic irradiance of 2500 μmol/m^2^/s for 1 s. The maximum quantum yield (Fv/Fm) was determined to evaluate the physiological status of mosses. The results are shown in Table 1.

#### 2.4.4. Molecular Identification of Fungal Isolates

The isolated fungal strains were identified at the genus levels based on the nucleotide sequences of the internal transcribed spacer region (ITS; ITS1–5.8S rRNA–ITS2). The ITS region was amplified using the primer pair ITS1 (5′-TCCGTAGGTGAACCTGCGG-3′) and ITS4 (5′-TCCTCCGCTTATTGATATGC-3′). PCR amplification was performed in a total volume of 25 µL. The thermal cycling conditions were as follows: initial denaturation at 98 °C for 3 min; 30 cycles of denaturation at 98 °C for 30 s, annealing at 54 °C for 30 s, and extension at 72 °C for 1 min; and a final extension at 72 °C for 5 min. PCR products were purified using a PCR Cleanup Kit and subjected to Sanger sequencing. Consensus sequences were aligned and compared against the NCBI GenBank database using the BLAST algorithm (https://blast.ncbi.nlm.nih.gov/Blast.cgi, accessed on 22 January 2026). Isolates were identified to the genus level when query coverage ≥ 95% and sequence identity ≥ 98.7%. All obtained sequences were deposited in the NCBI GenBank database under the accession numbers listed in Table 2.

#### 2.4.5. Preparation of Biocides

Three biocides (Total Algae Control, benzalkonium chloride, and dodecyl dimethyl benzyl ammonium chloride 1227) were prepared by serial dilution with sterile deionized water. The original commercial biocides were used as stock solutions and diluted to designed working concentrations using sterile deionized water. For fungal inhibition zone assays, the three biocides were diluted to final concentrations of 0.5%, 1%, 3%, and 5% (*v*/*v*). For algal growth inhibition tests, the biocides were adjusted to 1%, 3%, and 5% (*v*/*v*). For bryophyte killing tests, working solutions of 1%, 3%, and 5% (*v*/*v*) were prepared. All diluted biocide solutions were filtered through a 0.22 μm sterile membrane filter to eliminate microbial contamination and stored at room temperature in the dark prior to use.

#### 2.4.6. Algal Growth Inhibition Assay

Algae at logarithmic phase (OD_680_ = 0.05–0.1) were used. In 2 mL algal suspension, 200 μL biocide (1%, 3%, 5%) was added; CK received 200 μL deionized water. After 24 h, OD_680_ was recorded. Inhibition rate (IR) was calculated as:IR (%) = [(OD_control – OD_treatment)/OD_control] × 100% [22,23,24].

### 2.5. Fungal Inhibition Zone Assay (Oxford Cup Method)

Purified lichen-associated fungal mycelia were collected, homogenized, and suspended to prepare uniform fungal suspensions, which were evenly spread on the surface of PDA plates. The oxford cup method was used for antifungal assessment, with each cup loaded with 100 µL of biocide solution at gradient concentrations of 0.5%, 1%, 3%, and 5% (*v*/*v*). Sterile deionized water was applied as the solvent blank control. All treatments, including blank control and biocide groups, were performed in triplicate. The plates were incubated at 25 °C. Inhibition zone radii were measured quantitatively.

### 2.6. In-Situ Experiment

**Figure 2:**
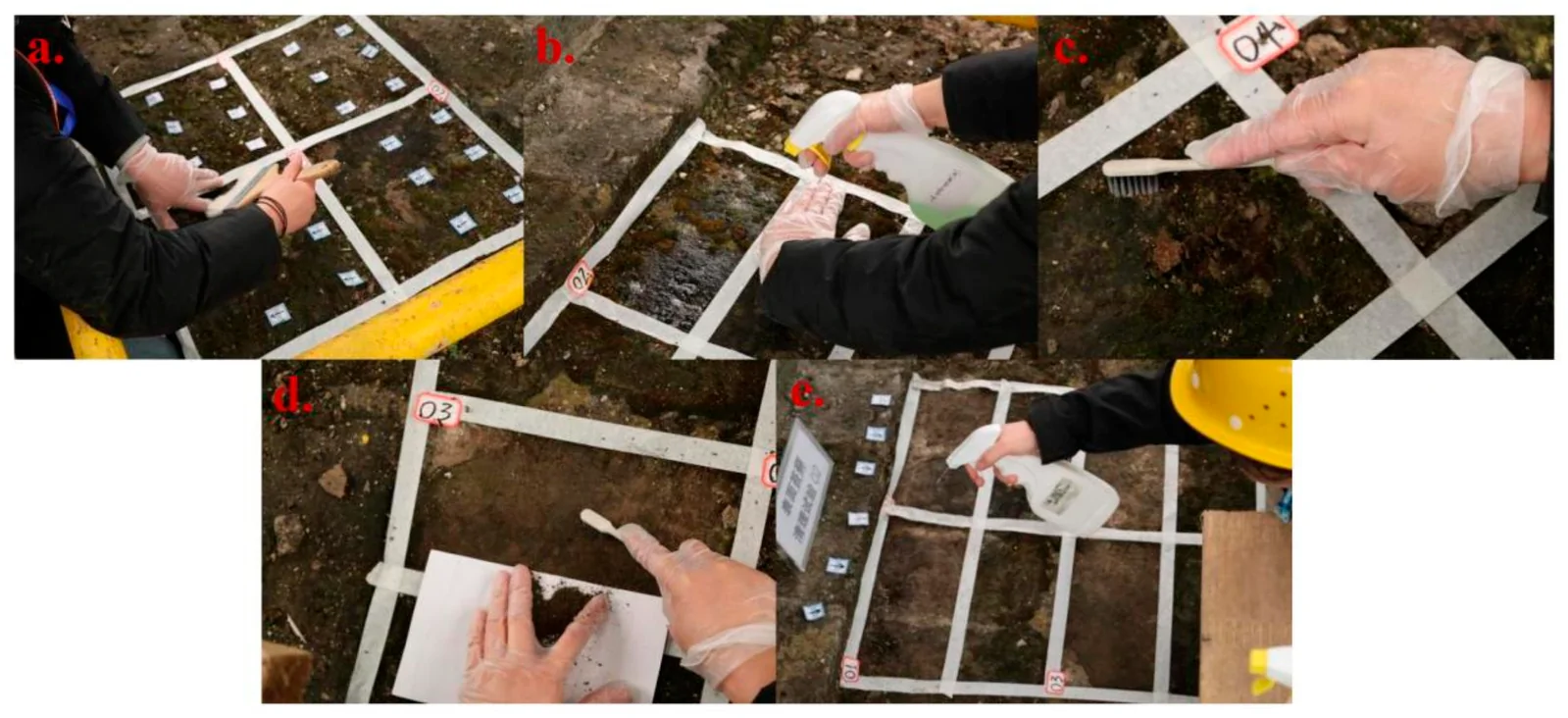
The in-situ biocide treatment procedure, including (**a**) pre-treatment cleaning, (**b**) first spraying, (**c**) residual removal, (**d**) water rinsing, and (**e**) re-spraying of biocides at 3% concentration.

In this study, four areas with vigorous algal growth were randomly selected on 16S 16S DNA Pagoda. Within each selected area, a rectangular experimental plot with a fixed length and width was established, which was divided into one control group and three treatment groups for subsequent experiments. First, a spatula or wooden tool was used to carefully remove prominent microbial coverings (such as moss, lichen, and mold) from the surfaces of the selected areas to reduce biomass, followed by gently brushing away loose contaminants with a soft-bristled brush (Figure 2a). Metallic tools were avoided to prevent scratching the stone surface. Subsequently, all three biocides, namely benzalkonium chloride, Total Algae Control, and dodecyl dimethyl benzyl ammonium chloride 1227, were applied at a uniform concentration of 3% and separately sprayed onto the designated experimental areas. Spraying was performed in the order from less polluted areas to more polluted areas to prevent cross-contamination (Figure 2b). The biocides were allowed to remain on the stone surface for 6 h to ensure their full efficacy, and the microbial killing effect was closely monitored. Thereafter, non-metallic scrapers were used to remove residual microbial tissues and biocide residues from the stone surface (Figure 2c), followed by gentle cleaning of the surface with a soft-bristled brush dipped in distilled water (Figure 2d). The three biocides at the same 3% concentration were sprayed again to enhance their inhibitory effect (Figure 2e). To evaluate the efficacy of the three biocides in algal removal and inhibition, a portable colorimeter was used to monitor the changes in algal color on the stone surface at 3 days, 7 days, 21 days and 30 days after the treatment. The color parameters measured by the colorimeter were used as quantitative indicators to reflect the degree of algal removal and the stability of the inhibitory effect [13].

## 3. Results and Discussion

### 3.1. Micromorphological Characteristics of Bryophytes

Ultra-depth three-dimensional microscopic observation of bryophyte samples 01 and 05 collected from Longhu Pagoda revealed that leaf cells were closely arranged in a typical single-layer structure, with intact and uniformly distributed chloroplasts, indicating vigorous physiological activity.

**Figure 3:**
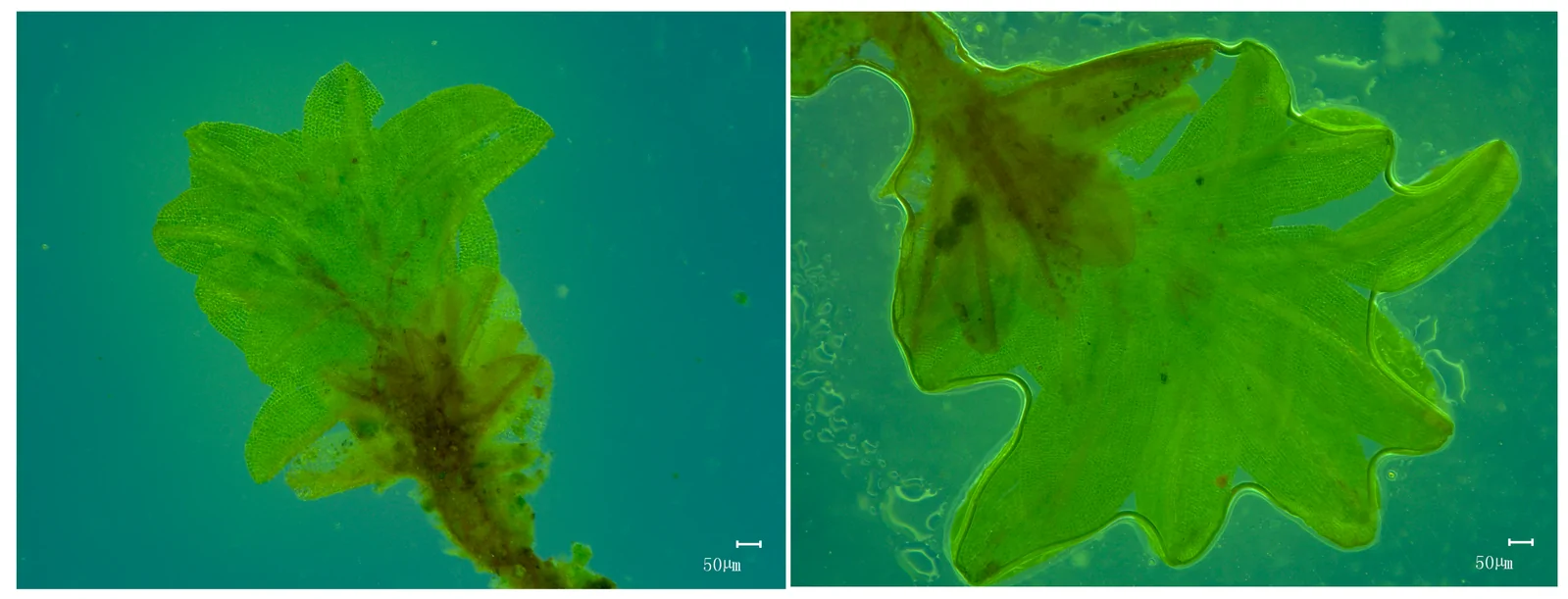
Micrograph of bryophyte sample 01 (200×).

**Figure 4:**
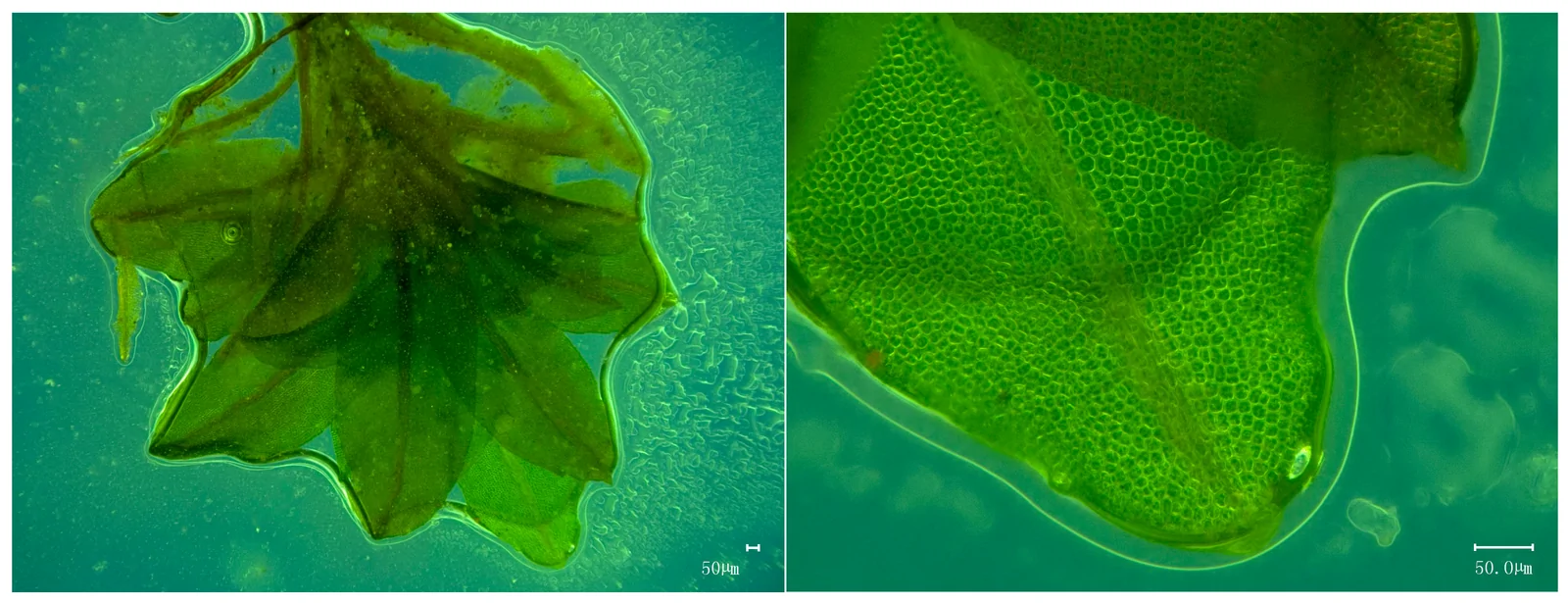
Micrographs of bryophyte sample 05 (100×, 500×).

**Figure 5:**
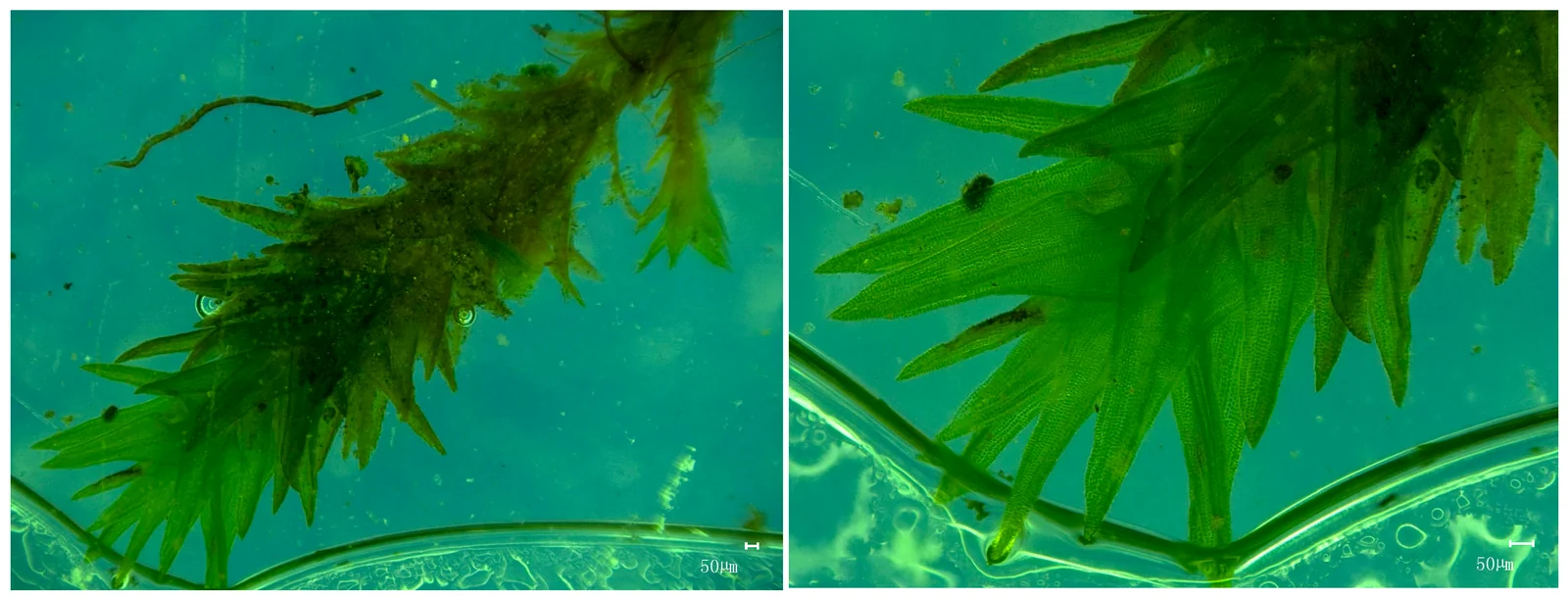
Micrographs of bryophyte sample 05 (100×, 200×).

According to the morphological criteria specified in *Bryologia Sinica*, the moss samples displayed in Figure 3 and Figure 4 are characterized by elongated hexagonal leaf cells and distinct leaf costae, which are consistent with the typical morphological features of *Bryum argenteum* Hedw. Furthermore, the sample in Figure 5 presents well-developed rhizoids, smaller cell size and thickened cell walls, exhibiting prominent xerophytic morphological traits analogous to species of the families *Pottiaceae* and *Grimmiaceae*.

### 3.2. Bryocidal Effects of Biocides

Wild-collected bryophytes generally exhibit asynchronous growth status, heterogeneous genetic backgrounds, and extensive surface microbial contamination. These inherent variations obstruct the establishment of a standardized toxicological evaluation framework and prevent the precise quantification of biocide inhibitory efficacy against mosses at different developmental stages. To guarantee the reproducibility and horizontal comparability of subsequent bryocidal experiments, pure spores of three commercially available and ecologically representative moss species were germinated under sterile laboratory conditions. The selected model species include *Bryohaplocladium microphyllum* (Hedw.) R. Watan. & Iwats. (common name: short velvet moss), *Bryum argenteum* Hedw. (common name: duoduo moss), and *Plagiomnium acutum* (Lindb.) T. J. Kop. These standardized moss materials were adopted as experimental organisms to simulate the stress tolerance characteristics of mature wild moss communities in natural stone habitats.

As a robust and widely employed technique, chlorophyll fluorescence analysis serves as a powerful tool for investigating the impacts of various stress factors on plant photosynthetic processes. Since its initial application, the Fv/Fm ratio has been widely adopted as a highly sensitive indicator for assessing plant photosynthetic capacity. A reduction in this parameter is generally indicative of impaired efficiency of photosystem II (PSII), a phenomenon commonly referred to as photoinhibition. Specifically, the maximum quantum yield of PSII (Fv/Fm) reflects the maximum efficiency of light energy conversion in photosynthetic organisms, with the following reference criteria: 0.75–0.85 (healthy plants), 0.60–0.70 (mild stress), <0.50 (severe stress/near death), and near zero (complete death) [25,26]. Therefore, this study employed chlorophyll fluorescence technology to evaluate the moss-killing efficacy of benzalkonium chloride, total Algae Control, and dodecyl dimethyl benzyl ammonium chloride 1227, and the experimental results are presented in Table 1.

Table 1 presents the Fv/Fm values of three moss species (*Microdus*, *Bryum*, and *Plagiomnium*) under different concentrations of the three test agents, along with their corresponding physiological statuses. Two physiological states were classified according to chlorophyll fluorescence characteristics. Structural death refers to irreversible damage to cell and chlorophyll structures with no detectable fluorescence signal, leading to a complete loss of recovery ability. Functional death is defined as Fv/Fm values close to zero; although cell structure and chlorophyll are preserved, photosynthetic activity is fully inhibited, and bryophytes cannot regrow or recolonize.

The control group (CK) exhibited Fv/Fm values ranging from 0.80 to 0.85, which falls within the reference range for healthy plants (0.75–0.85) [27,28], indicating that all moss species were in a healthy physiological state before the experiment, ensuring the reliability and validity of the subsequent efficacy evaluation.

With respect to benzalkonium chloride, no fluorescence signal was detected in *Bryohaplocladium microphyllum* and *Plagiomnium acutum* under all tested concentrations (5%, 3%, and 1%), which may be attributed to the severe damage to the photosynthetic system of these two moss species caused by the agent, leading to the complete loss of photosynthetic capacity. For *Bryum argenteum*, the Fv/Fm values were 0.47, 0.35, and 0.48 under 5%, 3%, and 1% benzalkonium chloride concentrations, respectively, all of which were less than 0.50, corresponding to severe stress or near death. Further observation showed that 2/3 of the Bryum individuals under each concentration exhibited structural death, while the remaining 1/3 were in a state of severe stress, suggesting that benzalkonium chloride has a strong inhibitory effect on the photosynthetic system of Bryum, but its efficacy varies among different moss species.

Total Algae Control showed distinct moss-killing efficacy at different concentrations. Under the 5% concentration, no fluorescence signal was detected in *Bryohaplocladium microphyllum* and *Plagiomnium acutum*, and the Fv/Fm value of Bryum was 0.47 (severe stress/near death), with 2/3 of *Bryum argenteum* individuals showing structural death and 1/3 in severe stress. When the concentration was reduced to 3% and 1%, no fluorescence signal was detected in all three moss species, indicating that the ketone-based inhibitor at concentrations ≤ 3% can cause complete structural death of the tested moss species, demonstrating stronger moss-killing efficacy than benzalkonium chloride at the same concentration.

Dodecyl dimethyl benzyl ammonium chloride 1227 exhibited the most potent moss-killing efficacy among the three agents. Under the 5% and 3% concentrations, the Fv/Fm values of all three moss species were between 0.07 and 0.09, which were near zero, indicating functional death of the mosses. Even at the lowest concentration (1%), the Fv/Fm values of *Bryohaplocladium microphyllum* and *Bryum argenteum* were still near zero (0.09 and 0.08, respectively), corresponding to complete death, while *Plagiomnium acutum* showed an Fv/Fm value of 0.27 (severe stress/near death). This indicates that dodecyl dimethyl benzyl ammonium chloride 1227 can effectively damage the photosynthetic system of mosses even at low concentrations, and its efficacy is relatively consistent across different moss species.

**Figure 6:**
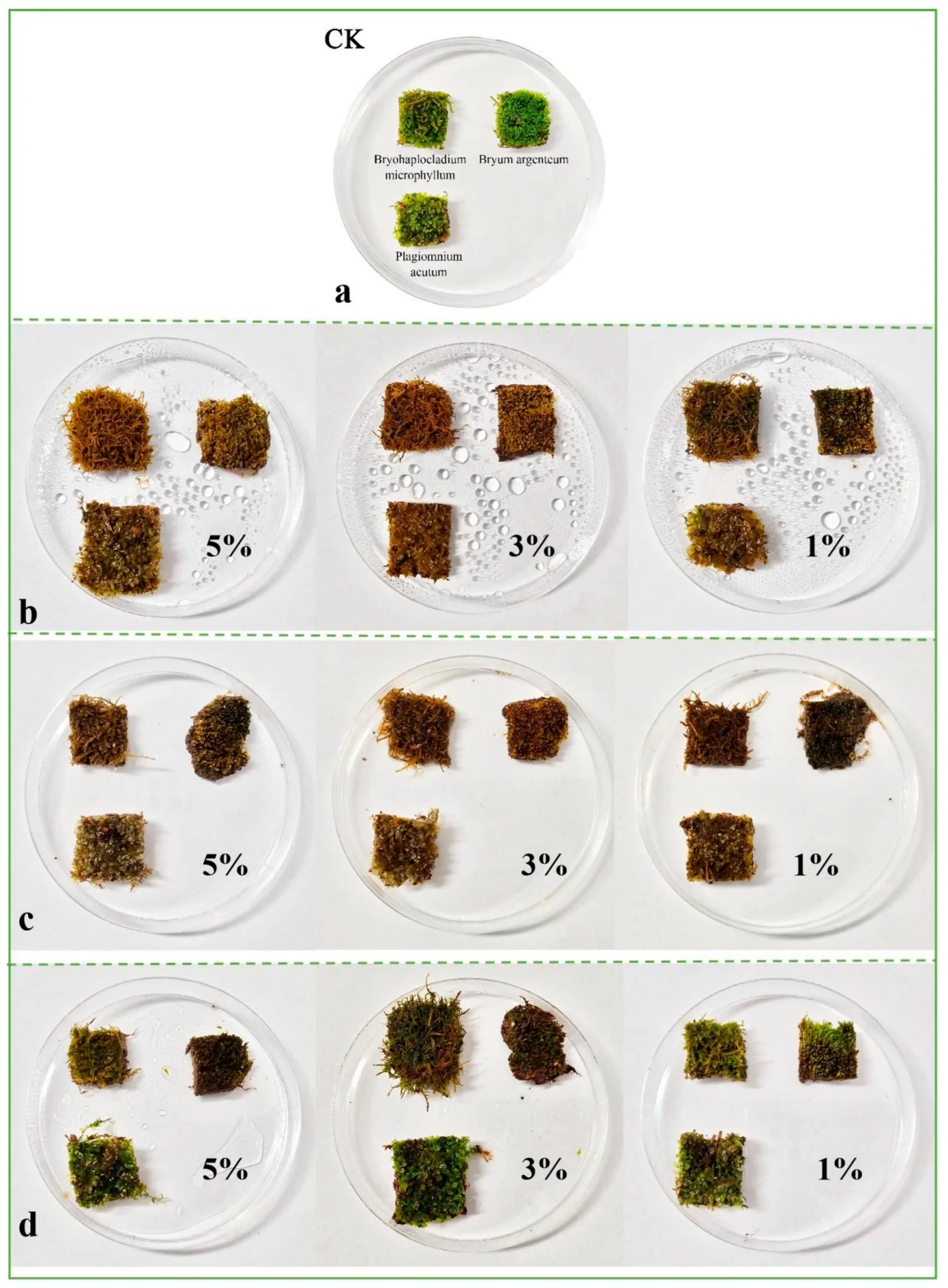
Bryocidal effects of three standard mosses treated with biocides for 24 h: (**a**) Control; (**b**) Benzalkonium chloride; (**c**) Total Algae Control; (**d**) Dodecyl dimethyl benzyl ammonium chloride 1227).

Figure 6 Bryocidal effects of three standard moss species (*Bryohaplocladium microphyllu*, *Bryum argenteum*, *Plagiomnium acutum*) treated with three biocides (benzalkonium chloride, ketone-based inhibitor, dodecyl dimethyl benzyl ammonium chloride 1227) at different concentrations (5%, 3%, 1%) for 24 h. The morphological damage (browning, wilting, structural necrosis) of mosses is highly consistent with the Fv/Fm values in Table 1, verifying the photosynthetic system injury and moss-killing efficacy of each biocide.

In summary, the Fv/Fm ratio, as a sensitive indicator of PSII efficiency, effectively reflects the damage degree of the three test agents to the moss photosynthetic system, and thus can be used to evaluate their moss-killing efficacy. Among the three agents, dodecyl dimethyl benzyl ammonium chloride 1227 showed the strongest moss-killing efficacy, followed by the ketone-based inhibitor, while benzalkonium chloride had relatively weak efficacy and significant species-specific differences. These results provide a scientific basis for the selection and application of moss-killing agents in practical scenarios.

### 3.3. Isolation and Identification of Lichen Symbiotic Algae and Fungi

**Figure 7:**
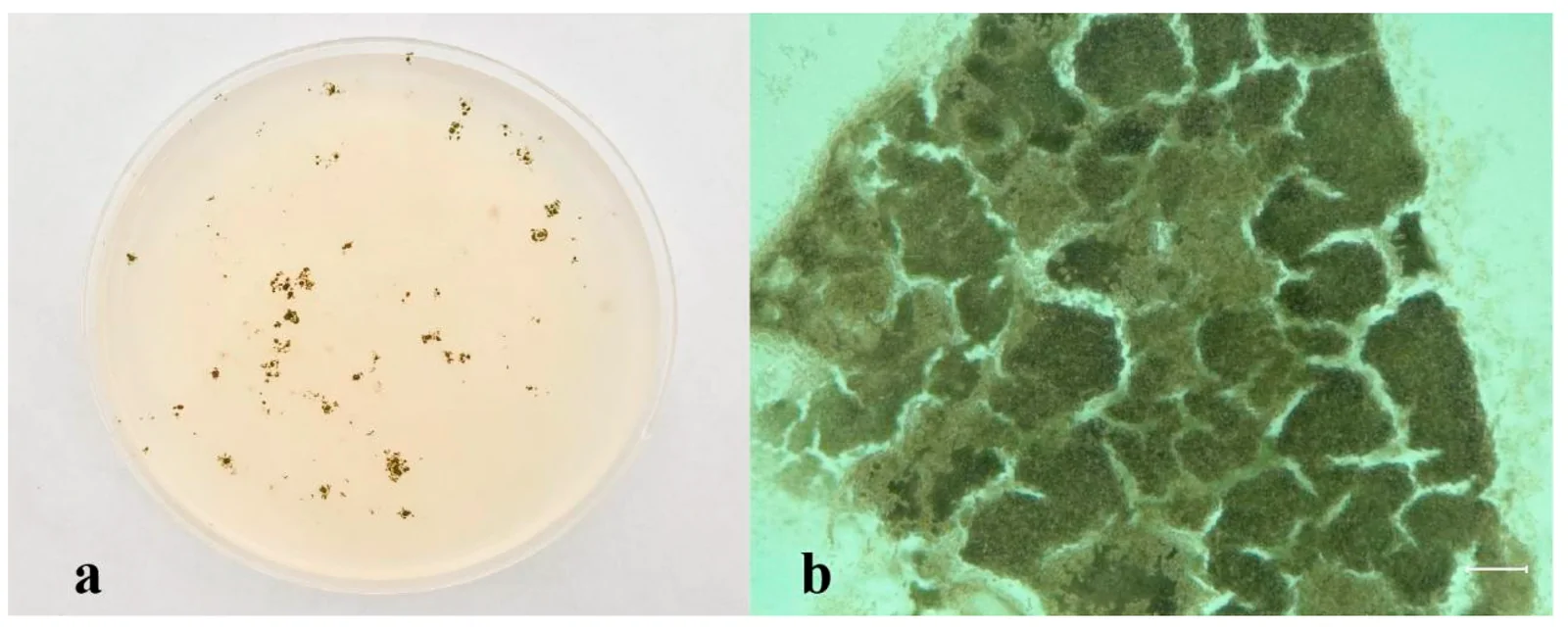
Isolation and morphological characterization of symbiotic algae from lichen samples. (**a**) Axenic algal colonies formed on BG11/BBM solid medium after 1 week of culture. (**b**) Light micrograph of *Chlorella* sp. (eukaryotic green alga, lichen symbiont), scale bar = 50 μm.

As shown in Figure 7, symbiotic algae were successfully isolated and purified from the lichen samples of Longhu Pagoda samples 02, 03, and 04. After one week of cultivation on BG11 and BBM solid media, distinct and uniform algal colonies were observed (Figure 7a). Following purification and enrichment in liquid medium, a stable axenic strain was obtained, which was identified as *Chlorella* sp. Based on light microscopic observations (Figure 7b). Specifically, the colonies of this strain consisted of spherical to subspherical unicellular microalgae with a diameter of approximately 3–10 μm, which is consistent with the typical morphological characteristics of green algae *Chlorella*. These algal cells were non-motile, contained well-developed chloroplasts, and often aggregated into small clusters embedded in a gelatinous matrix, serving as the dominant eukaryotic photosynthetic symbionts in the lichens of Longhu Pagoda.

**Figure 8:**
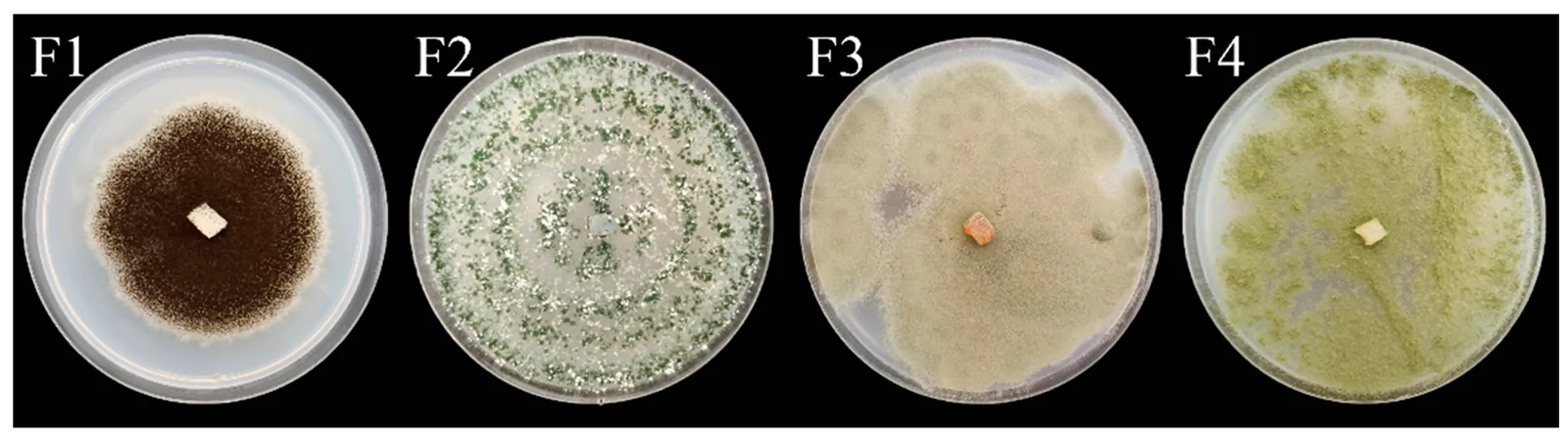
Morphologies of four isolated fungal strains (F1–F4) from lichen samples.

Four distinct fungal strains (F1–F4) were successfully isolated from the lichen thalli collected from Longhu Pagoda, and their macroscopic morphological characteristics on PDA medium after 7 days of incubation at 25 °C are shown in Figure 8. Strain F1 produced dense, dark brown to blackish colonies with a velvety texture radiating from the center of the plate, which is typical of *Aspergillus niger*. Strain F2 formed greenish, floccose colonies with a white mycelial base, characteristic of *Trichoderma yunnanense*. Strain F3 grew as fast-spreading, light beige to off-white colonies with a powdery texture, consistent with the morphology of *Talaromyces ruber*. Strain F4 developed yellowish-green, velvety colonies with a radial growth pattern, matching the colonial features of *Aspergillus aflatoxiformans*. Molecular identification based on ITS sequence analysis (Table 2) confirmed the above morphological assignments.

Combined morphological and molecular results confirmed that the dominant lichen-associated fungi on Longhu Pagoda belong to the genera *Aspergillus*, *Trichoderma*, and *Talaromyces*. Among them, *Aspergillus* species (F1, F4) are well-documented biodeteriogens on stone cultural heritage, capable of producing organic acids that accelerate the dissolution of carbonate and silicate minerals in stone substrates. *Trichoderma yunnanense* (F2) and *Talaromyces ruber* (F3) are also common saprophytic fungi in outdoor environments, which can colonize stone surfaces, secrete extracellular enzymes, and synergistically exacerbate the biodeterioration of cultural relics. These findings provide a direct biological basis for the subsequent screening of targeted biocides for the protection of Longhu Pagoda.

### 3.4. Growth Inhibition Assay of Lichen Symbiotic Algae

The growth inhibitory effects of the three biocides on the tested algae were evaluated by measuring the optical density at 680 nm (OD_680_) after 24 h of treatment, and the results are summarized in Table 3.

The growth inhibitory effects of three biocides on the tested algae were assessed by measuring OD_680_ after 24 h of treatment, with results presented in Table 3. All three biocides showed concentration-dependent growth inhibition on the lichen symbiotic algae. With increasing biocide concentration from 1% to 3%, algal biomass (OD_680_) gradually decreased and inhibition rates correspondingly increased. Among the three agents, dodecyl dimethyl benzyl ammonium chloride 1227 exhibited the strongest inhibitory activity, with inhibition rates ranging from 54.5% to 63.7%. Total Algae Control showed moderate inhibition (41.8–51.8%), whereas benzalkonium chloride displayed the weakest effect, with inhibition rates below 45% at all concentrations. These results indicate that dodecyl dimethyl benzyl ammonium chloride 1227 has superior potential for controlling the growth of lichen symbiotic algae. These results provide a scientific basis and data support for the selection and application of suitable algicidal agents in the biodeterioration control and conservation of the Longhu Pagoda.

### 3.5. Antifungal Activity of Biocides (Inhibition Zone Assay)

**Figure 9:**
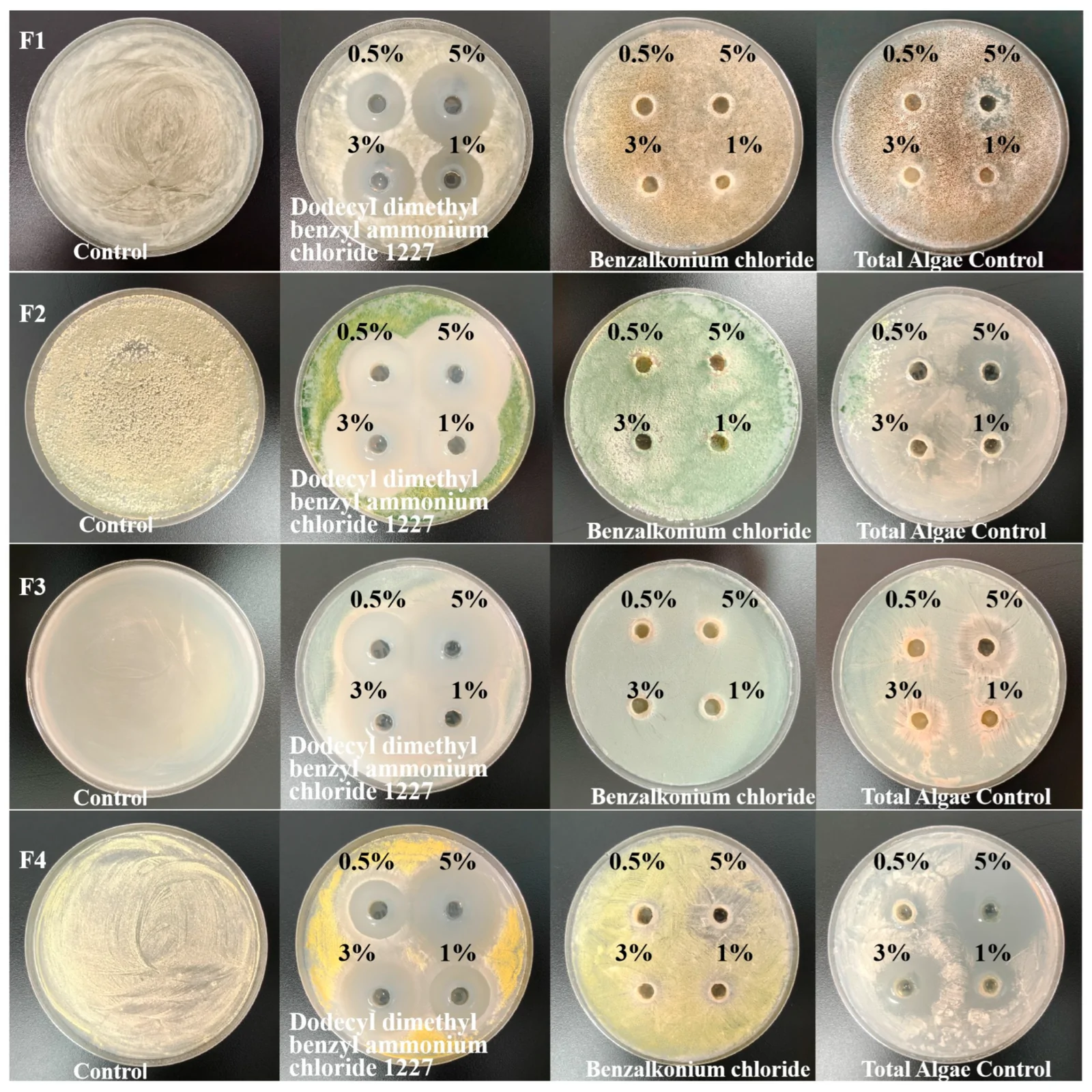
Inhibition zones of three biocides against fungal strains F1–F4.

The antifungal performance of three biocides against four fungal strains (F1–F4) isolated from Longhu Pagoda was evaluated by the inhibition zone assay, as visually presented in Figure 9 and quantitatively summarized in Table 4. All values are shown as mean ± SD (n = 3). Dodecyl dimethyl benzyl ammonium chloride 1227 exhibited the strongest and broad-spectrum antifungal activity, generating clear inhibition zones against all tested strains at all concentrations (0.5–5%). The inhibition zones displayed a significant concentration-dependent increase. At 5% concentration, the inhibition zone radii reached 1.292 cm for F1, 1.879 cm for F2, 1.936 cm for F3, and 1.474 cm for F4, respectively, consistent with the distinct transparent inhibition zones observed in Figure 9. The Total Algae Control inhibitor showed moderate antifungal activity, with visible inhibition zones only at concentrations of 1% and above. Benzalkonium chloride displayed the weakest and most strain-specific antifungal effect. No inhibition zones were detected against F1, F2, and F3 at any concentration. Only at 5% concentration did benzalkonium chloride produce a weak inhibition zone (0.739 cm) against F4, which was also consistent with the visual results in Figure 9. Among the four fungal strains, F4 (*Aspergillus flavus*) was the most sensitive to all three biocides. In contrast, F1 (*Aspergillus niger*) and F2 (*Trichoderma yunnanense*) showed relatively higher tolerance, especially to benzalkonium chloride and Total Algae Control inhibitors at low concentrations. All effective biocides exhibited a clear dose–effect relationship, with larger inhibition zones at higher concentrations.

### 3.6. Evaluation of In Situ Treatments

**Figure 10:**
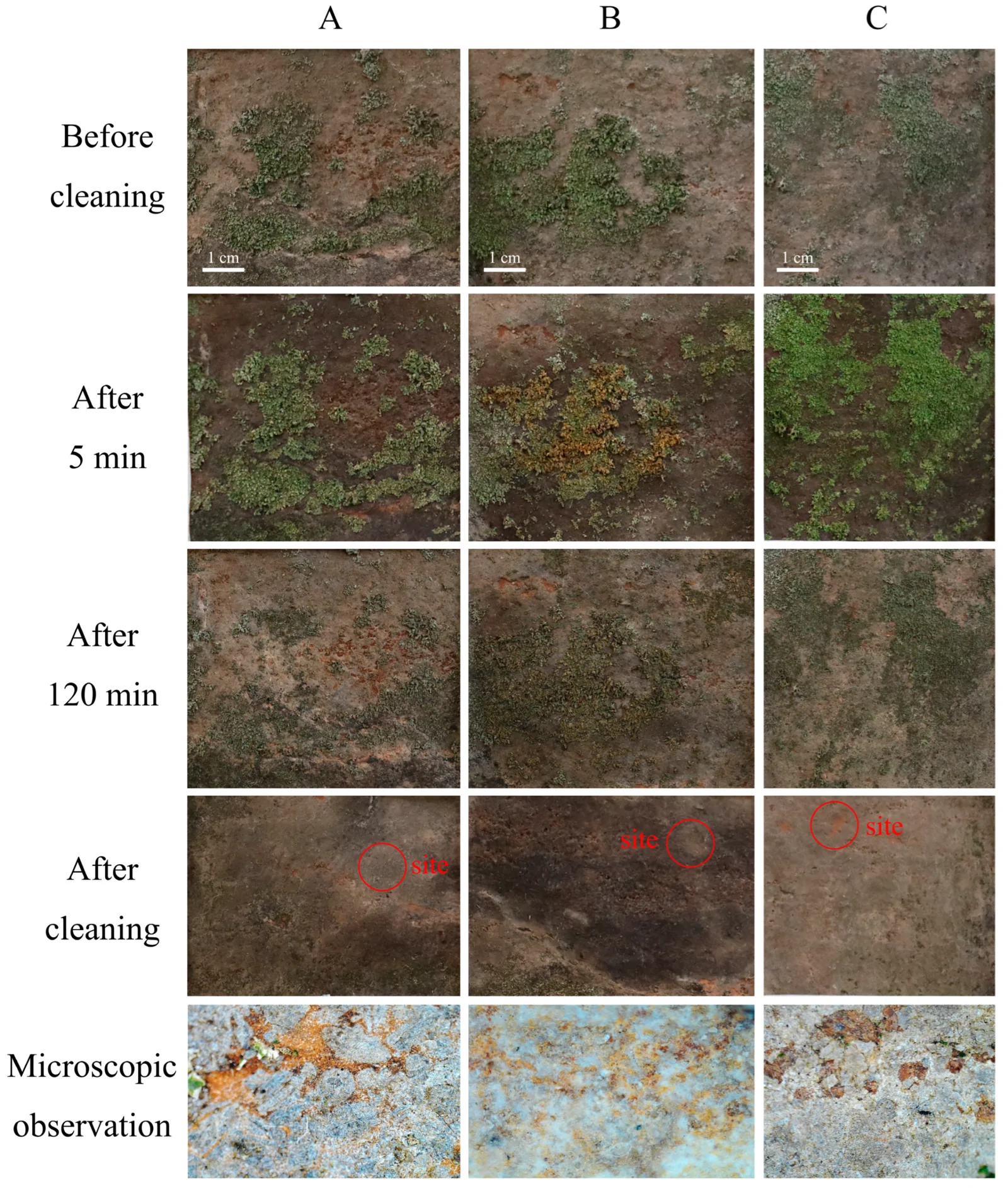
Biocidal effect of three biocides ((**A**) Benzalkonium chloride, (**B**) Total Algae Control, (**C**) Dodecyl dimethyl benzyl ammonium chloride 1227)) on stone biofilms, observed at different treatment stages (before cleaning, 5 min, 120 min, post-cleaning) and post-cleaning microscopic verification at 250× magnification. Red circles indicate microscopic sampling sites.

Figure 10 illustrated the in-situ biocidal effects of three biocides on stone biofilms, with time-series macroscopic observations and post-cleaning microscopic verification. For benzalkonium chloride (Group A), the moss showed no obvious discoloration after 5 min of treatment, and partial green residual was still observed after 120 min. Microscopic observation confirmed obvious biological residues, indicating slow onset and incomplete killing effect, which was consistent with its species selectivity in vitro. Total Algae Control (Group B) exhibited the most excellent performance: the moss turned brown rapidly within 5 min, achieved complete inactivation after 120 min, and the stone surface was completely restored after cleaning. Microscopic observation showed no intact biological structures, demonstrating the fastest onset and the most thorough killing effect, which was highly consistent with the structural death observed in laboratory tests. Dodecyl dimethyl benzyl ammonium chloride 1227 (Group C) showed good immediate effect at 5 min and complete inactivation at 120 min, with excellent final cleaning effect and only trace degradation residues in microscopic observation, matching its functional death performance in vitro. The field results, verified by microscopic analysis, fully confirmed the reliability of the laboratory evaluation system, and provided a scientific basis for the selection of moss control agents for stone cultural heritage conservation.

**Figure 11:**
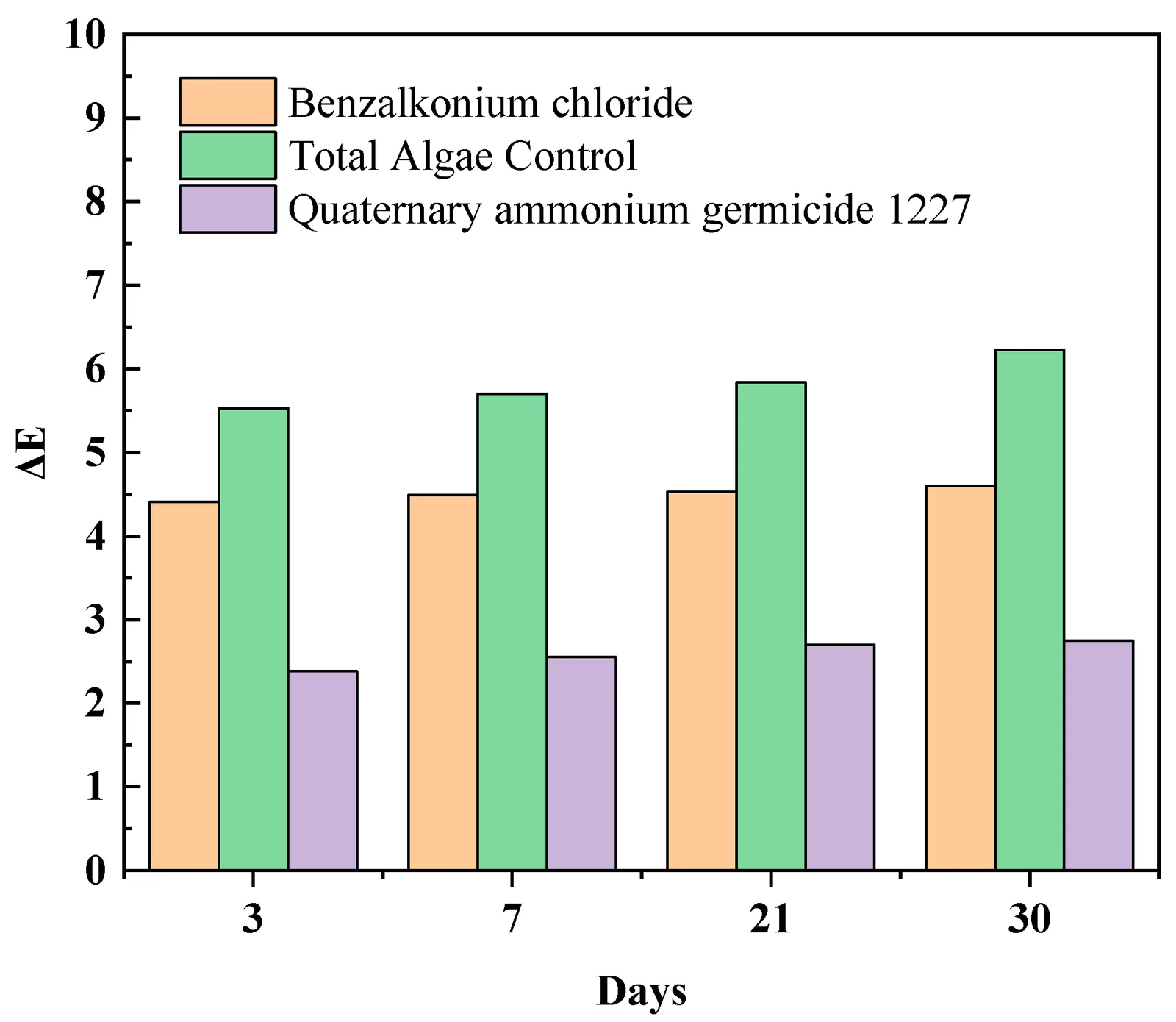
Long-term color difference (ΔE) of stone samples treated with three biocides (benzalkonium chloride, Total Algae Control, and dodecyl dimethyl benzyl ammonium chloride 1227) monitored at 3, 7, 21, and 30 days after treatment.

As shown in Figure 11, the ΔE values of all three biocides remained stable during the 30-day monitoring period. Dodecyl dimethyl benzyl ammonium chloride 1227 exhibited the lowest ΔE values (2.3–2.8), indicating the minimal color impact on the stone substrate and excellent compatibility. Benzalkonium chloride showed moderate ΔE values (4.4–4.6), while Total Algae Control presented the highest ΔE values (5.5–6.2), suggesting a more significant color change to the stone surface. Furthermore, a protective shelter has been constructed around Longhu Pagoda in practical conservation projects, which blocks direct rainfall and reduces stone surface humidity. This measure effectively cuts off the water source for algal and bryophyte regrowth and greatly reduces the risk of microbial recolonization. Overall, dodecyl dimethyl benzyl ammonium chloride 1227 satisfies the requirements for stone cultural relics protection with favorable color stability and substrate compatibility. Nevertheless, this study has certain limitations. The evaluation is based only on short-term monitoring data, lacking long-term verification of the sustained efficacy and substrate stability of biocides on stone materials under field conditions. Additionally, the potential environmental impacts and ecological risks of the tested biocides have not been systematically assessed. Future work will focus on long-term field monitoring of biocide durability and comprehensive environmental safety evaluation, so as to further improve the technical system for biodeterioration control of stone cultural relics.

## 4. Conclusions

This study focused on the biological deterioration of the open-air stone cultural relic Longhu Pagoda, and systematically investigated the community composition, micromorphological characteristics, and control efficacy of three typical biocides against bryophytes, lichen-associated fungi, and symbiotic algae. The dominant bryophytes on the pagoda surface were identified as members of *Bryum argenteum* Hedw, *Pottiaceae* and *Grimmiaceae*. Four typical stone-biodeteriorating fungi were isolated and identified, namely *Aspergillus niger* (F1), *Trichoderma yunnanense* (F2), *Talaromyces ruber* (F3) and *Aspergillus aflatoxiformans* (F4). The three biocides showed distinct moss-killing mechanisms: benzalkonium chloride and ketone-based inhibitor caused chlorophyll degradation and structural death, whereas dodecyl dimethyl benzyl ammonium chloride 1227 induced functional death by blocking photosynthesis without damaging chlorophyll. Among all tested agents, dodecyl dimethyl benzyl ammonium chloride 1227 exhibited the strongest and broadest inhibitory activity against algae and fungi, followed by ketone-based inhibitor, while benzalkonium chloride displayed the weakest and most species-specific effect. The experimental protocols and evaluation methods adopted in this study are fully replicable for the prevention and control of biological diseases in similar open-air stone cultural heritage sites. For engineering applications, dodecyl dimethyl benzyl ammonium chloride 1227 is recommended as the preferred biocide. Necessary precautions include conducting preliminary compatibility tests on inconspicuous stone areas before large-scale use, and avoiding excessive contact with porous or colored stone structures to ensure surface safety. Comprehensive evaluation confirmed that dodecyl dimethyl benzyl ammonium chloride 1227 exhibits high efficiency and broad-spectrum performance while preserving stone appearance, rendering it a highly appropriate biocide for mitigating biological deterioration on Longhu Pagoda. This work provides scientific basis and technical support for mitigating biodeterioration at comparable open-air stone cultural heritage sites.

## Figures and Tables

**Table 1:** Maximum quantum yield (Fv/Fm) of mosses under different treatments.

Group	Concentration	*Bryohaplocladium microphyllum*	*Bryum argenteum*	*Plagiomnium acutum*	Status
CK	-	0.8	0.85	0.81	Healthy
Benzalkonium chloride	5%	-	0.47	-	Structural death (2/3) Severe stress (1/3)
	3%	-	0.35	-	Structural death (2/3) Severe stress (1/3)
	1%	-	0.48	-	Structural death (2/3) Severe stress (1/3)
Total Algae Control	5%	-	0.47	-	Structural death (2/3) Severe stress (1/3)
	3%	-	-	-	Structural death
	1%	-	-	-	Structural death
Dodecyl dimethyl benzyl ammonium chloride 1227	5%	0.08	0.08	0.07	Functional death
	3%	0.07	0.07	0.09	Functional death
	1%	0.09	0.08	0.27	Near death

Note:“-” represents ‘No Signal’, indicating that the instrument failed to detect the fluorescent signal (below the detection limit), which suggests extremely low or complete degradation of chlorophyll content.

**Table 2:** Molecular identification of fungal strains isolated from Longhu Pagoda.

Strain	Closest Relative in GenBank	Query Cover (%)	Identity (%)	Accession No.
F1	*Aspergillus niger* CBS 121.28	95	100	NR_163668.1
F2	*Trichoderma yunnanense* CBS 121219	97	99.45	NR_134419.1
F3	*Talaromyces ruber* CBS 132704	99	99.63	NR_111780.1
F4	*Aspergillus aflatoxiformans* CBS 143679	95	99.27	NR_171606.1

**Table 3:** OD_680_ and inhibition rates of algae after 24 h treatment.

Group	Concentration	*Chlorella* sp.	
		OD680	IR (%)
CK	-	0.0910	-
Benzalkonium chloride	1%	0.0540	40.7
	3%	0.0515	43.4
	5%	0.0504	44.6
Total Algae Control	1%	0.0530	41.8
	3%	0.0433	52.4
	5%	0.0439	51.8
Dodecyl dimethyl benzyl ammonium chloride 1227	1%	0.0414	54.5
	3%	0.0344	62.2
	5%	0.0330	63.7

**Table 4:** Inhibition zone radii (cm) of biocides against fungal strains. Values are mean ± SD (n = 3).

Biocide	Conc.	Ring Radius			
		F1	F2	F3	F4
Benzalkonium chloride	5%	-	-	-	0.739 ± 0.010
	3%	-	-	-	-
	1%	-	-	-	-
	0.5%	-	-	-	-
Total Algae Control	5%	0.644 ± 0.015	0.856 ± 0.048	0.732 ± 0.053	1.496 ± 0.013
	3%	0.427 ± 0.009	0.463 ± 0.017	0.505 ± 0.036	0.855 ± 0.038
	1%	-	-	0.367 ± 0.003	0.588 ± 0.024
	0.5%	-	-	-	0.413 ± 0.007
Dodecyl dimethyl benzyl ammonium chloride 1227	5%	1.292 ± 0.014	1.879 ± 0.016	1.936 ± 0.023	1.474 ± 0.012
	3%	1.056 ± 0.017	1.573 ± 0.030	1.668 ± 0.030	1.233 ± 0.030
	1%	0.755 ± 0.025	1.238 ± 0.035	1.221 ± 0.010	0.831 ± 0.020
	0.5%	0.690 ± 0.034	1.081 ± 0.022	0.975 ± 0.015	0.681 ± 0.021

Note: “-” = no visible inhibition zone.

## Data Availability

The original contributions presented in this study are included in the article. Further inquiries can be directed to the corresponding author.
